# Herpes Simplex Virus Evasion of Early Host Antiviral Responses

**DOI:** 10.3389/fcimb.2019.00127

**Published:** 2019-04-30

**Authors:** Eduardo I. Tognarelli, Tomás F. Palomino, Nicolás Corrales, Susan M. Bueno, Alexis M. Kalergis, Pablo A. González

**Affiliations:** ^1^Millennium Institute on Immunology and Immunotherapy, Departamento de Genética Molecular y Microbiología, Facultad de Ciencias Biológicas, Pontificia Universidad Católica de Chile, Santiago, Chile; ^2^Departamento de Endocrinología, Facultad de Medicina, Escuela de Medicina, Pontificia Universidad Católica de Chile, Santiago, Chile

**Keywords:** interferon (IFN), inflammasome, toll-like receptors (TLRs), natural killer cells (NK cells), dendritic cells (DCs), cytosolic nucleic acid receptors, innate immunity, apoptosis

## Abstract

Herpes simplex viruses type 1 (HSV-1) and type 2 (HSV-2) have co-evolved with humans for thousands of years and are present at a high prevalence in the population worldwide. HSV infections are responsible for several illnesses including skin and mucosal lesions, blindness and even life-threatening encephalitis in both, immunocompetent and immunocompromised individuals of all ages. Therefore, diseases caused by HSVs represent significant public health burdens. Similar to other herpesviruses, HSV-1 and HSV-2 produce lifelong infections in the host by establishing latency in neurons and sporadically reactivating from these cells, eliciting recurrences that are accompanied by viral shedding in both, symptomatic and asymptomatic individuals. The ability of HSVs to persist and recur in otherwise healthy individuals is likely given by the numerous virulence factors that these viruses have evolved to evade host antiviral responses. Here, we review and discuss molecular mechanisms used by HSVs to evade early innate antiviral responses, which are the first lines of defense against these viruses. A comprehensive understanding of how HSVs evade host early antiviral responses could contribute to the development of novel therapies and vaccines to counteract these viruses.

## Introduction

Herpes simplex viruses (HSVs) type 1 (HSV-1 or human herpesvirus 1, HHV-1) and type 2 (HSV-2 or human herpesvirus 2, HHV-2), are members of the *Herpesviridae* family and *Alphaherpesvirinae* subfamily, similar to varicella zoster virus (VZV) (Davison, 2010; Sharma et al., 2016). HSVs are present among humans at a high prevalence (Looker et al., 2008; CDC, 2010; Yawn and Gilden, 2013; Dickson et al., 2014; Suazo et al., 2015b), with two thirds of the global population infected with HSV-1 (Looker et al., 2015a), and ~11% of the world population infected with HSV-2 (Looker et al., 2015b). HSV-1 and HSV-2 are associated with diverse clinical manifestations, yet disease widely varies from one individual to another, with nearly 40% of those that are infected displaying symptoms during primary infection (Langenberg et al., 1999; Bernstein et al., 2013). Additionally, during recurrent viral reactivations, most individuals are asymptomatic, with 5–15% of those infected displaying clinical symptoms related to HSV infections (Benedetti et al., 1994; Wald et al., 2000; Sudenga et al., 2012; Suazo et al., 2015b). Although a relatively low proportion of the infected individuals show clinical manifestations, the high percentage of the world population infected with these viruses yields an enormous number of individuals that effectively suffer from HSV-related illnesses.

HSV-1 is mainly associated with orofacial lesions, yet it is also the leading cause of infectious blindness in developed countries and the number one cause of viral encephalitis in adults (Kaye and Choudhary, 2006; Horowitz et al., 2010; Farooq and Shukla, 2012; Bernstein et al., 2013). On the other hand, HSV-2 is mainly associated with genital lesions and neonatal encephalitis (Gupta et al., 2007; Berger and Houff, 2008; Looker et al., 2008; Suazo et al., 2015b), despite the fact that HSV-1 is nowadays more frequently related to primary genital infection worldwide (Buxbaum et al., 2003; Coyle et al., 2003; Xu et al., 2006; Pereira et al., 2012). However, HSV-2 reactivates more frequently from the genital tissue than HSV-1 and hence, despite the finding that the latter is commonly detected during primary infection, HSV-2 is more often isolated from this site than HSV-1 at any time during infection (Lafferty et al., 1987; Kaneko et al., 2008). A similar phenomenon may occur in the orofacial area, with most viral reactivations being attributed to HSV-1. Variable reactivation of HSV-1 and HSV-2 from neurons within the trigeminal or sacral ganglia may be given by differences in gene expression profiles by neurons that innervate these tissues (Kaneko et al., 2008; Flegel et al., 2015; Lopes et al., 2017).

A clinically relevant concern associated with HSV-2 genital infection is that it is associated with a three-fold increase in the likelihood of acquiring the human immunodeficiency virus type 1 (HIV-1), due to synergistic aspects related to the co-infection with both viruses (Wasserheit, 1992; Freeman et al., 2006; Barnabas et al., 2011). For instance, evidence of an indirect interplay between HIV and HSV occurs with HSV-2 infection of macaques and humans eliciting an increase in the amounts of dendritic cells present in the genital tissue, as well as α_4_β_7_- and CCR5-expressing CD4^+^ T cells, both known to be substrates for HIV (Rebbapragada et al., 2007; Martinelli et al., 2011). HSV-2 also elicits lesions in the infected tissue that provide an entry portal for HIV (Bagdades et al., 1992; Suazo et al., 2015b). Additionally, proposed interactions between HSV-2 and HIV would support HSV-2 infections being associated with a relative risk of HIV incidence nearing 50% in the African region (Looker et al., 2017). The association between HSV-2 and HIV suggests that tackling HSV-2 infection could help reduce the HIV pandemics (Rebbapragada et al., 2007; De Jong et al., 2010; Johnson et al., 2011; Martinelli et al., 2011; Sartori et al., 2011; Stefanidou et al., 2013a). Therefore, HSV-2 infection should be considered a major matter of public health concern.

Infections with HSVs remain latent and are characterized by sporadic reactivation episodes accompanied by virus shedding, regardless of the presence of clinical symptoms (Kaneko et al., 2008; Tobian et al., 2013). Lifelong infection in the host by HSVs is achieved thanks to their capacity to infect neurons, mainly those enervating infected tissues and then remain latent within these cells (Margolis et al., 2007; Yao et al., 2014). In the skin, mucosae and eyes, HSVs access neurons by infecting sensorial nerve termini and then traveling in a retrograde manner through the axon of these cells up to the soma. Later, HSVs may reactivate from these cells and exit them through anterograde movements either, to infect other neurons that eventually may innervate the brain or infect cells located nearby the initial site of infection (Linehan et al., 2004; Gonzalez and Sanjuan, 2013).

Importantly, HSVs not only infect epithelial cells and neurons but virtually any cell type in the body, including immune cells thanks to the fact that the main receptors of HSVs are widely distributed in host tissues and cells (Krummenacher et al., 2004). By infecting immune cells, these viruses can modulate and escape diverse antiviral mechanisms evolved by the host to counteract infection and furthermore, establish long-term infection with sporadic recurrences that produce new infectious particles (Retamal-Diaz et al., 2015; Suazo et al., 2015a). Here, we review and discuss recent studies that report the relationship between HSVs and early cellular antiviral responses, both in immune and non-immune cells.

### Replication Cycle of HSVs

**Figure 1:**
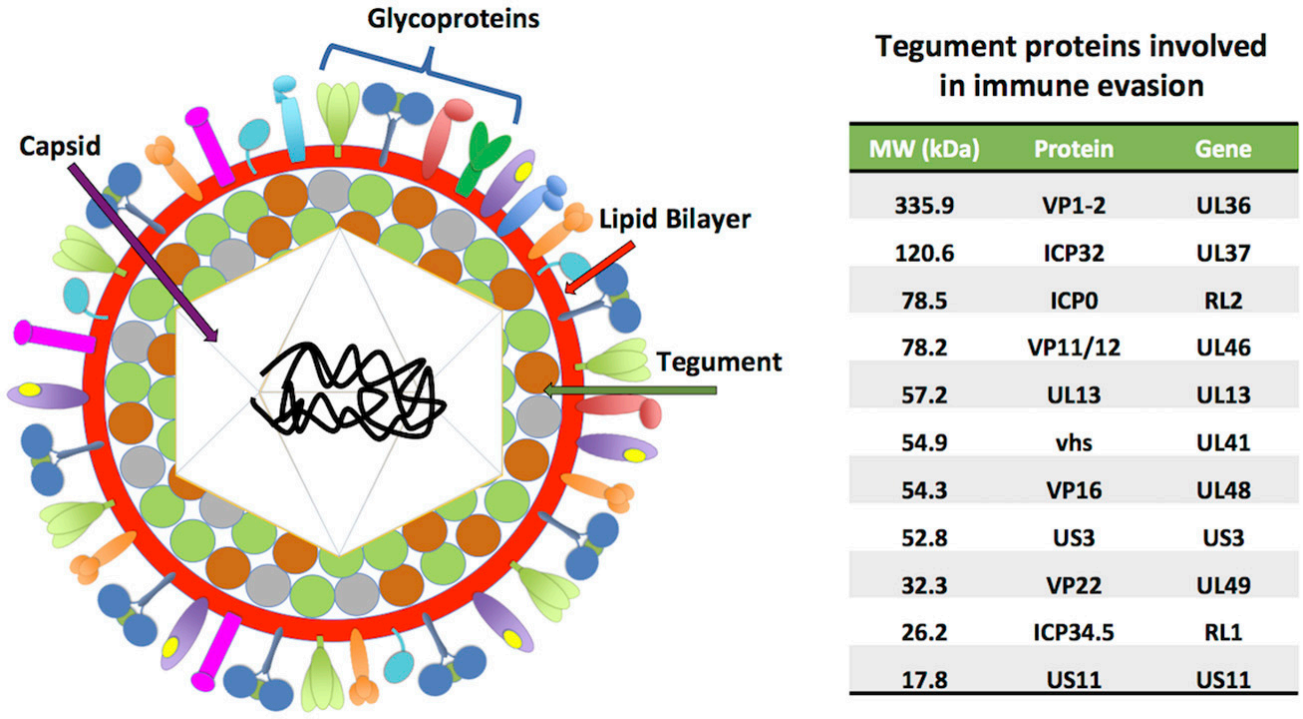
HSV virion structure. HSVs possess linear, double-stranded DNA genomes (152–154 kbp) encoding more than 70 ORFs. The viral genomes are contained within icosahedral capsids of ~125 nm, which in turn are surrounded by complex meshes of viral proteins known as the tegument. The tegument is enveloped by lipid membranes, which harbor numerous transmembrane glycoproteins. A table with tegument proteins involved in immune evasion is shown on the right, ordered from highest to lowest molecular weight (MW).

HSV-1 and HSV-2 share ~74% identity at the nucleotide level and are structurally very closely related (Baines and Pellett, 2007). Both viruses have a linear, double-stranded DNA (dsDNA) genome with sizes ranging from 150 to 154 kbp, which encode more than 70 open reading frames (ORFs) (Kieff et al., 1971; Dolan et al., 1998; Koelle et al., 2017). The viral genomes are covered by a 125 nm icosahedral capsid (Wu et al., 2016), which is surrounded by a mesh composed by many proteins (>20) called the tegument (Figure 1). This protein stratum is in turn enveloped by a lipid bilayer, from which multiple viral glycoproteins protrude and play roles in virus entry and exit, as well as immune-modulation and immune-escape (Roller and Roizman, 1992; Loret et al., 2008; Retamal-Diaz et al., 2015; Suazo et al., 2015a; Suk and Knipe, 2015).

Although HSV-1 and HSV-2 share common aspects during cell entry, they do have some differences. For instance, unlike HSV-1, HSV-2 does not require its glycoprotein C (gC) for attaching to target cells (Shukla and Spear, 2001). On the other hand, both of these HSVs do require the viral glycoprotein B (gB) for the virus to attach to heparan sulfate proteoglycans on the cell surface (Atanasiu et al., 2013). In immune cells such as dendritic cells (DCs) and natural killer cells (NK cells), gB has been reported to bind to an additional cell receptor for viral attachment, namely the paired immunoglobulin like-type 2 receptor (PILR) (Shiratori et al., 2004; Satoh et al., 2008). Once the virus has attached to the cell surface, the viral glycoprotein D (gD) will bind to either, nectin-1 (or nectin-2) expressed on the surface of most anchored cells in the organism, such as epithelial and neuronal cells, or the herpesvirus entry mediator (HVEM), a member of the tumor necrosis factor receptor (TNFR) family that signals intracellularly depending on the orientation of its ligand, either in *cis* or *trans* (Kovacs et al., 2009). The latter HSV receptor is preferentially expressed on the surface of immune cells, such as DCs and T cells (Krummenacher et al., 2004; Jones et al., 2016). In addition, gD has been described to bind to 3-*O*-sulfated heparan sulfates on the surface of CHO cells, which permitted viral entry when gB, gD, glycoprotein H (gH), and glycoprotein L (gL) were present in the virion (Xia et al., 2002; Tiwari et al., 2004). As a result of gD binding to its ligand, this glycoprotein will undergo a conformational changes that enable this protein to activate the viral gH/gL glycoprotein complex, which in turn triggers the fusion of the virus and cell membranes in a process that is dependent on the activity of gB, which acts as the fusion protein for these viruses (Lazear et al., 2014). Moreover, the glycoprotein complex gH/gL of HSV-1 and HSV-2 participate in a process distinct to the conventional viral entry, since they have been reported to bind αv3, αvβ6 and αvβ8 surface integrins causing dissociation from the heterodimer permitting gH activation to promote virion entry through a mechanism involving acidic endosomes (Gianni et al., 2013, 2015; Cheshenko et al., 2014). Lastly, it has also been observed that HSV-1 can enter cells via a phagocytosis-like uptake mechanism (Clement et al., 2006).

Once the viral and cell membranes have fused, the viral capsid, which is surrounded by tegument proteins, will be released into the cytoplasm. These tegument proteins will have the opportunity to rapidly modulate host antiviral determinants upon entry into the cell, interfering with the detection of viral components, that altogether aim at diminishing the progression of infection (Owen et al., 2015). Within the cytoplasm, the viral capsid will associate to microtubules and travel toward the nuclear membrane to deliver the viral genome into the cell nucleus (Sodeik et al., 1997; Dohner et al., 2002; Radtke et al., 2010). However, it is possible that the capsid reaches the nucleus by simple diffusion within the cytoplasm, as morphological changes take place in HSV-infected cells (Ibanez et al., 2018). Once the capsid reaches the outer nuclear membrane, the tegument viral protein VP1/2, which travels associated to the capsid, will anchor this structure to nuclear pore proteins and favor its docking to the nucleopore for the injection of the viral DNA into the nucleus. This process will allow the initiation of viral gene transcription within the nucleus, and later on, viral genome replication (Abaitua et al., 2012). In parallel, the viral protein VP16, which is present in the tegument, will localize in the nucleus in such a way to promote the transcription of viral genes, acting as a transactivator (Milbradt et al., 2011; Roizman and Zhou, 2015; Suk and Knipe, 2015).

Within infected cells, HSV genes are transcribed sequentially in three main waves; the first set of viral genes that are transcribed are called *immediate early* (or alpha) genes, with many of their functions being related to limiting host immediate antiviral mechanisms. This set of genes also encodes proteins that act as transcription factors that promote the transcription of the second set of viral genes (Silva et al., 2012). After the transcription of alpha genes, *early* (or beta) viral genes are expressed, which are involved among others in promoting the replication of the viral genome (Ibanez et al., 2018). During the replication of the viral genetic material, the genomes of HSVs undergo circularization in a form known as “*rolling circle*,” which is regulated by viral factors that ultimately generate linear genomes that are packaged into new viral capsids within the nucleus (Jackson and Deluca, 2003). After the expression of alpha and beta genes, HSV-infected cells transcribe *late* (or gamma) viral genes, which are occasionally separated into *late early* (or gamma-1) and *late* (or gamma*-2*) genes, and are involved among others in providing the structural components that are present in the virion (Chen et al., 1992). During viral transcription, host cells equipped with a zinc-finger antiviral protein (ZAP) that can utilize this restriction factor to inhibit the replication of viruses by promoting the degradation of critical viral mRNAs. Nevertheless, the HSV-1 UL41 protein which is also known as the virus host shutoff protein (VHS), has been reported to rapidly degrade human ZAP mRNA upon infection, before this host factor can block viral gene expression (Su et al., 2015).

Importantly, infectious HSV particles to be released from the infected cells will require that the viral capsids leaving the nucleus contain the viral genome. For this, HSV capsids are assembled with the viral DNA within the nucleus and then envelope in the inner nuclear membrane (INM) and de-envelope from the outer nuclear membrane (ONM) (Mettenleiter et al., 2013; Funk et al., 2015). At this time, tegument proteins coating the capsid are acquired both, in the nucleus and cytoplasm. Importantly, viral glycoproteins located within the perinuclear space are needed to carry out the capsid budding and fusion processes with these membranes (Bucks et al., 2007; Mou et al., 2009; Ott et al., 2011; Mettenleiter et al., 2013; Owen et al., 2015). Once in the cytoplasm, the capsid which will be covered with tegument proteins will be enveloped into the Golgi apparatus, generating enveloped particles within this compartment that are ready for virion exocytosis (Johnson and Baines, 2011). Notably, it has been reported that viral glycoproteins acquired by the enveloped capsid in the Golgi apparatus are first exported to the cell surface by this organelle and then re-internalized through the Trans Golgi Network before associating to the coated capsids (Wisner and Johnson, 2004; Turcotte et al., 2005). Infected cells will attempt to prevent the release of mature virions using the host antiviral restriction factor tetherin, an interferon (IFN)-inducible membrane protein that has been shown to prevent egress of several enveloped viruses (Perez-Caballero et al., 2009; Kuhl et al., 2011). However, the HSV-1 VHS protein depletes tetherin by degrading its mRNA (Zenner et al., 2013), while, HSV-2 gD has been observed to directly interact with a long disulfide-rich coiled-coil structure (CC) that is found within the extracellular domain of tetherin, thereby targeting the latter to lysosomes for degradation (Liu et al., 2016b). Both effects evidence how HSVs intervene with cell antiviral mechanisms meant to stop virions exit and prevent dissemination.

Aside from the previously described events, HSVs can propagate onto close cells through cell-cell interactions. In these cases, viral proteins are directed to the interface of adjacent cells in a process termed virological synapse, in which cells in close proximity undergo membrane fusion events favoring virus propagation (Johnson et al., 2001). An advantage of this type of infection is that it allows HSVs to propagate onto neighboring cells while avoiding being targeted by immune components, such as complement or neutralizing antibodies (Hook et al., 2006a; Lubinski et al., 2011). This mechanism of infection has not only been reported for epithelial cells, but also for the infection of immune cells, such as T cells by HSV-infected fibroblasts (Aubert et al., 2009).

In sum, HSVs have evolved molecular determinants to effectively bind to and infect various cell types, causing productive infection in multiple tissues and establishing latency in neurons. Alternatively, these viruses are also capable of infecting immune cells and modulate their functions to further interfere with host early antiviral responses.

### HSV Modulates Apoptosis Differentially in Non-immune and Immune Cells

**Figure 2:**
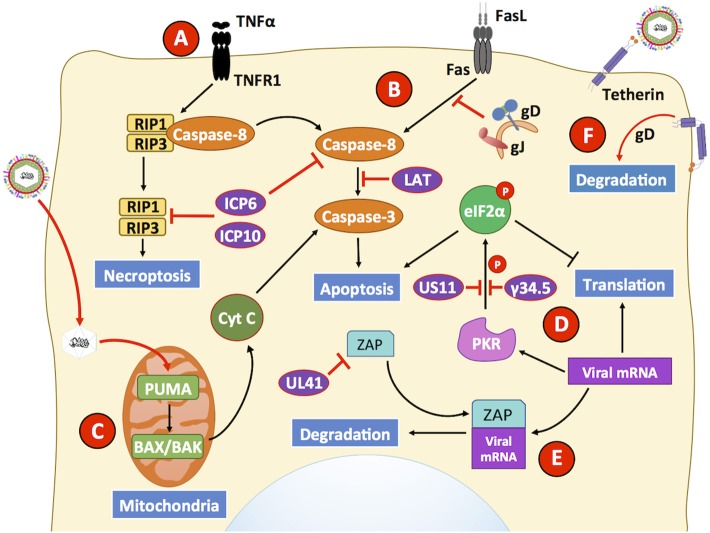
HSVs modulate antiviral mechanisms related to cell death in non-immune cells. HSVs utilize numerous mechanisms to hamper the capacity of host cells to restrict viral infection. **(A)** The engagement of the TNFR receptor leads to the activation of caspase-8 eliciting apoptosis, or eventually RIP1/3 to induce necroptosis. However, HSV proteins ICP6 and ICP10 hamper signaling events related to these pathways, thus prolonging cell survival during infection. **(B)** Engagement of the Fas receptor with Fas ligand (FasL) generally leads to extrinsic apoptosis events mediated by the activation of caspase-8. However, HSV glycoproteins J (gJ) and gD block signaling events by this receptor. Additionally, the LAT transcript also interferes with caspase-8 mediated signaling that usually leads to apoptosis. **(C)** However, HSV infection has been described to upregulate the expression of PUMA in the mitochondria of HSV-infected cells, which leads to BAX/BAK-dependent apoptosis mediated by caspase-3. Thus, HSV may induce the intrinsic apoptotic pathway at later time points of infection after inhibiting apoptosis. **(D)** Another antiviral mechanism hampered by HSV infection is inhibition of cell-induced apoptosis due to translation arrest. Upon detection of viral components, host PKR triggers eIF2α phosphorylation, which inhibits its function and consequently mRNA translation, leading to global protein synthesis arrest and caspase-3 activation. However, the viral proteins US11 and γ34.5 impair eIF2α phosphorylation, allowing viral gene translation to ensue during infection and limiting apoptosis through this pathway. **(E)** The host protein ZAP can act as an antiviral factor that promotes degradation of viral mRNAs. However, its function is inhibited by the HSV protein UL41 (VHS), which promotes ZAP mRNA degradation. **(F)** Finally, infected cells may attempt to prevent the release of mature virion through a membrane protein called tetherin that is capable of binding to enveloped virions. As a countermeasure, the viral glycoprotein gD interacts with tetherin which ultimately provokes degradation of the latter. Black lines show cellular processes. Red lines show processes modulated by HSVs.

For a virus to produce significant amounts of infectious particles from an infected cell, it will need the cell to be viable for as long as possible and to provide the building blocks required for replicating its genetic material and producing its proteins. HSVs have been reported to modulate cellular death in different cell types either, to promote cell viability for the generation of new virions or to promote the death of cells that may be detrimental for their replication and shedding. For instance, the HSV-1 glycoproteins J (gJ) and D (gD) have been described to produce, at least partially the inhibition of Fas-mediated apoptosis in a neuroblastoma cell line and Jurkat cells (Zhou et al., 2000; Jerome et al., 2001). Surprisingly, the expression of gJ alone also induced the production of reactive oxygen species (ROS), which can eventually trigger apoptosis (Figure 2) (Fleury et al., 2002; Aubert et al., 2008). HSV-1 has also been reported to reduce cell apoptosis in epithelial cells, despite eliciting processes that involved FLICE-inhibitory protein (c-FLIP) downregulation, which is an inhibitor of caspase-8 that generally results in cell death (Kather et al., 2010). This apparent discrepancy was attributed to the presence of latency-associated transcript (LAT) sequences, which have been described to act as inhibitors of caspase-8-mediated apoptosis, similar to what occurs in infected neuronal cells (Henderson et al., 2002).

Additionally, an intrinsic mechanism of apoptosis consists on the activity of pro-apoptotic Bcl-2 cell death in mouse fibroblasts and monocytes, as well as in human colon carcinoma cells (Figure 2) (Sciortino et al., 2006; Papaianni et al., 2015). Importantly, HSV-1 infection promotes increased expression of p53 upregulated modulator of apoptosis (PUMA), a protein that is a host Bcl-2 homology 3 (BH3)-only family member that activates Bax/Bak and produces mitochondrial outer membrane permeabilization (MOMP) to release cytochrome c from the mitochondria and activates caspase-3, ultimately culminating in apoptosis (Papaianni et al., 2015). Furthermore, during HSV infection, caspase-8-interacting domains within the HSV-1 viral protein ICP6 and the HSV-2 viral protein ICP10, both which are R1 large subunits of a ribonucleotide reductase (RR), have been suggested to bind to caspase-8 and cause inhibition of apoptosis induced by TNF-α-induced apoptosis through the TNF receptor TNFR1 (Figure 2) (Guo et al., 2015a). However, this inhibition of apoptosis may cause cells to enter necroptosis 12 h post-infection, as an alternative defense mechanism to limit virus replication and spreading (Sridharan and Upton, 2014). Nevertheless, HSV R1 proteins have been reported to bind to host receptor-interacting protein (RIP) 1/3 and inhibit necroptosis in human cells, while necroptosis was observed in mouse cells (Guo et al., 2015b; Huang et al., 2015). RIP3 likely mediates necroptosis in infected fibroblasts cells to limit the dissemination of HSV-1 in the mouse, similar to what has been described for RIP3 with other viruses, such as vaccinia virus and murine cytomegalovirus (MCMV) (Wang et al., 2014; Huang et al., 2015). Effective inhibition of both, apoptosis- and necroptosis-related mechanisms likely allow these viruses to generate high virus yields and sufficient amounts of progeny virions for the dissemination of infection onto adjacent cells and other tissues within the host.

On the other hand, HSVs have been described as capable of inducing apoptosis in immune cells (Jones et al., 2003; Stefanidou et al., 2013a). For instance, HSV-1 induces apoptosis in natural killer cells (NK cells) upon interacting with virus-infected macrophages that expresses Fas/FasL (Figure 2) (Iannello et al., 2011), and kills dendritic cells (discussed in the followings sections) (Peretti et al., 2005; Stefanidou et al., 2013b). Although the specific mechanism by which HSVs induce apoptosis in DCs is unclear to date, the process was found to be likely mediated by reduced c-FLIP expression, because it was targeted to degradation in a proteasome-dependent manner (Kather et al., 2010; Stefanidou et al., 2013a). Importantly, an HSV-2 mutant virus lacking the gene that encodes glycoprotein D (*US6*), was shown to be non-lethal for DCs, yet it is unknown if the mutated or deleted viral gene is directly involved in cell death or if its deletion interferes with viral processes that relate to cell death (Petro et al., 2015; Retamal-Diaz A. et al., 2017).

HSVs have also been described to induce the death of T cells either, directly or indirectly. Indeed, a study reported that HSV-2 induced apoptosis in T cells through the activation of caspase-9, -8, and -3 (Vanden Oever and Han, 2010). Although the mechanism by which apoptosis was induced involved intrinsic apoptotic pathways, the addition of inhibitors of apoptosis was unable to completely revert cell death (Pongpanich et al., 2004). Indirectly, HSV-1 has been described to induce T cell “fratricide,” a process in which activated T cells infected with HSV-1 increase their surface expression of FasL and induce the apoptosis of neighbor T cells, through FasL signaling through Fas receptor (Raftery et al., 1999). Overall, the findings discussed above indicate that HSVs can differentially modulate apoptosis in immune and non-immune cells, which may favor interference with the host antiviral immune response while allowing viral replication to occur in epithelial cells.

## HSVs Interfere With Toll-LIKE Receptor Sensing of Viral Components

Immune and non-immune cells express numerous molecular sensors that detect virus components or infection-related stimuli that promote the induction of rapid antiviral responses that hamper viral replication and virus propagation (Mogensen, 2009). A type of stimuli that may be encountered or produced during virus infection are pathogen-associated molecular patterns (PAMPs) (Tang et al., 2012), as well as danger signals released due to cellular stress in response to viral replication and known as damage-associated molecular patterns (DAMPs) (Johnson et al., 2013). Host receptors that sense these stimuli include Toll-like receptors (TLRs), which include both cytosolic and nuclear proteins (Mogensen, 2009). Upon the engagement of ligands by such types of receptors, signaling pathways take place which results in the expression of factors with antiviral activity, as well as the production of soluble and membrane-bound molecules that modulate the activity of the infected cell and neighboring cells (Pandey et al., 2014). The early recognition of viral factors by the host, immediately after infection will favor an effective control of the pathogen and hamper its replication and dissemination, altogether likely promoting the establishment of a protective and long-lasting immunity (Mogensen and Paludan, 2001; Tang et al., 2012).

Toll-like receptors, such as TLR2, TLR3, TLR7, and TLR9 have been described to mediate antiviral activities against HSVs during infection (Alexopoulou et al., 2001; Triantafilou et al., 2014). Experimental findings indicate that TLR2 recognizes glycosaccharides within the virion structure, which provides some degree of protection against HSVs. Indeed, it has been reported that TLR2 recognizes the glycoprotein B (gB) of HSV-1, promoting NF-κB activation and the secretion of interleukin (IL)-8 (Cai et al., 2013). Additionally, TLR2 seems to work in concert with the integrin αvβ3, acting as a coreceptor for its activation which leads to type-I IFN production in response to the HSV-1 proteins gH/gL (Gianni and Campadelli-Fiume, 2014). *In vivo* assays showed that in TLR2 knockout mice neuronal CCL2 levels were decreased, in association with reduced macrophage recruitment into the enteric nervous system after intragastric HSV-1 infection (Brun et al., 2018).

On the other hand, the use of agonists of TLR3, a receptor that recognizes pathogen or host double-stranded RNA (dsRNA) that may be produced during viral infections or abnormal cellular processes, has been reported to promote effective antiviral responses (Alexopoulou et al., 2001; Weber et al., 2006). Among HSV-related viral processes that occur during viral transcription, overlaps within (intra-molecular) or between (inter-molecular) viral and host mRNAs may yield dsRNA structures that induce the activation of dsRNA sensors. Additionally, HSVs encode micro RNAs (miRNAs, miR), which are processed from dsRNA intermediates. Some of these miRNAs have been shown to be involved in regulating virus latency. For instance, miR-H2 targets ICP0, which is required for immediate early gene expression and lytic infection, while miR-H3 and miR-H4 encode antisense sequences that counteract the neurovirulent virus lytic factor ICP34.5 (γ34.5). Furthermore, miR-H6 targets ICP4 and promotes LAT transcription (Piedade and Azevedo-Pereira, 2016). Other miRNAs, such as miR-H1, miR-H5, miR-H7, miR-H8, and miR-H11 are also loaded onto the RNA-induced silencing complex (RISC), which may also help trigger dsRNA sensors within infected cells (Flores et al., 2013). Although the precursors of these miRNAs may eventually be involved in the activation of host dsRNA sensors, the precise mechanisms by which these receptors are activated have not been determined and calls for further research in this area. Interestingly, the application of the TLR3 agonist Poly I:C was reported to confer protection against HSV-related disease in the mouse genital infection model (Ashkar et al., 2004). Recently, an HSV vaccine candidate based on sub-unit viral antigens used Poly I:C as a potent adjuvant, which elicited a robust antibody response and induced protection to a lethal vaginal challenge with HSV-2 in the mouse infection model. Importantly, protection was associated with the activation of TLR3 by this formulation (Bardel et al., 2016). On the other hand, it has been suggested that CD8α dendritic cells TLR3 expression contributes to the establishment of an antiviral response that is dependent on NK and CD8^+^ T cell activation (Swiecki et al., 2013).

Importantly, several findings suggest that the host has set mechanisms dependent on TLR3 function to detect HSV infection in the central neural system (CNS) and restrict viral replication (Zhang et al., 2008; Carty et al., 2014). For instance, experiments with TLR3 knockout mice have shown that the expression of TLR3 in astrocytes favors the control of HSV infection in the CNS, mainly thanks to NF-κB-dependent secretion of IL-6 and TNF-α (Reinert et al., 2012; Liu et al., 2013). On the other hand, induced pluripotent stem cells (iPSCs) obtained from TLR3-deficient patients that were differentiated into various neural populations, displayed increased susceptibility to viral infection and impaired IFN secretion (Lafaille et al., 2012). Accordingly, mutations present in genes of the TLR3 signaling pathway, such as the gene encoding for TANK-binding kinase 1 (TBK1), correlated with the development of herpes simplex encephalitis (HSE) in children and young adults (Herman et al., 2012; Lim et al., 2014). Therefore, positive modulation of the TLR3 pathway may help control HSV infection in infected individuals, yet this remains to be determined.

TLR7, which recognizes exogenous single-stranded RNA (ssRNA) has been reported to induce a response that reduces HSV infection and disease in a genital mouse infection model when engaged with the synthetic agonist Imiquimod (Miller et al., 1999). Furthermore, application of this TLR7 agonist in HIV-1-positive patients suffering from acyclovir-resistant HSV-2 disease has been shown to elicit favorable results against this virus. Thus, artificially engaging TLR7 during HSV-2 infection may eventually prove an effective mechanism to reduce virus-related disease and shedding in these patients (Hirokawa et al., 2011; Deza et al., 2015).

TLR9 is expressed in immune and non-immune cells and can sense bacterial and viral DNA, as well as synthetic CpG-oligodeoxynucleotides (CpG ODNs). Interestingly, intranasal application of CpG ODNs that are TLR9 agonists in BALB/c mice previous to HSV-1 infection was reported to reduce viral load and the production of pro-inflammatory cytokines IL-6, CCL2, and CCL5 by neurons in the CNS, which increased the survival rate of the infected mice (Boivin et al., 2012). Moreover, local mucosal TLR9 engagement with CpG ODNs prior to infection has been described to promote thickening of the genital epithelium and increase immune cell infiltration into the submucosa in order to control HSV-2 replication, conferring protection in the genital tissue after infection in mice (Ashkar et al., 2003). Although TLR9 knockout mice did not die after CNS infection with HSV-1 in one study, these animals were highly susceptible to HSV infection (Krug et al., 2004; Mancini and Vidal, 2018). In another study, TLR9 expression in the trigeminal ganglia was reported to be required to prevent HSV encephalitis induced by intranasal HSV-1 infection, as more than half of the animals that lacked this receptor died. Interestingly, if the animals lacked both, TLR2 and TLR9 all animals died after infection pointing out the relevance of these receptors in HSV infection (Lima et al., 2010). A similar result has been reported in the HSV genital infection model, as both TLR9 and TLR2 together have been observed to be relevant for resisting intravaginal infection by HSV-1. Indeed, a double TLR2/9 knockout mouse was more susceptible to infection than single knockout animals (Uyangaa et al., 2018). The anti-HSV response in the presence of TLR2/9 involved increased differentiation of TNF-α- and iNOS-producing DCs (Tip-DCs) and the activation of NK cells, which was accompanied by increased recruitment of the latter to the site of infection (Uyangaa et al., 2018). Furthermore, CpG treatment has been shown to induce plasmacytoid DCs (pDCs) to secrete IL-12 and type-I IFNs during HSV-2 infection in TLR4 knockout mice, but not TLR9 knockout animals suggesting that these cells need TLR9 to produce these cytokines (Lund et al., 2003; Boivin et al., 2012). Although IFN-α production during HSV infection *in vivo* is mostly TLR9-independent, CpG also elicited significant IFN-α secretion by splenic pDCs in a TLR9-dependent manner during HSV-1 infection *in vitro* (Hochrein et al., 2004).

Taken together, several TLRs recognize HSV components leading to limited disease, while other TLRs are not stimulated by HSV. However, when these receptors are engaged with activating ligands they also display antiviral activities, which suggests that targeting TLR receptors could be an attractive strategy to treat or limit HSV infection.

## HSVs Also Hamper the Sensing of Viral Nucleic Acids by non-TLR Receptors

**Figure 3:**
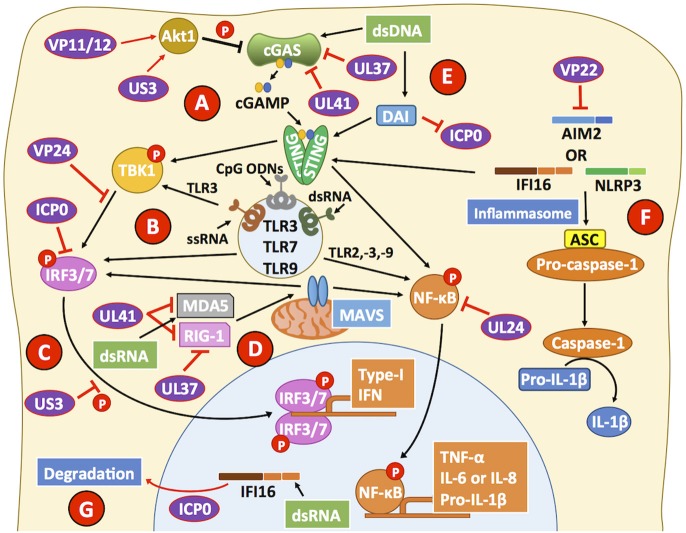
HSVs interfere with viral sensing. **(A)** cGAS is a cytosolic DNA sensor that triggers the activation of STING, which can lead to the phosphorylation of the transcription factor NF-κB and the transcription factor IRF3 through the activity of TBK1. HSV proteins, such as UL37 and UL41 interfere with cGAS activity. VP11/12 and US3 modulate Akt signaling to promote cGAS phosphorylation and suppress its activity, further impairing the capacity of cGAS to mediate STING activation. **(B)** Toll-like receptors (TLRs) are involved in recognizing pathogen and danger signals. Engagement of TLRs with agonists leads to improved antiviral responses due to increased type-I IFN secretion, which is dependent on IRF3/7 and leads to the production of cytokines dependent on NF-κB activation. Importantly, VP24 can target TBK1 to block IRF3 phosphorylation. Downstream of TBK1, ICP0 binds IRF3, and IRF7 to inhibit their activity. **(C)** US3 also blocks IRF3 activation and its translocation to the nucleus reducing type-I IFN production by HSV-infected cells. **(D)** MDA5 and RIG-1 can recognize dsRNA products elicited during viral infection and replication. HSV proteins UL37 and UL41 can impair the function of these cellular sensors, which signal through MAVS to activate NF-κB and promote cytokine production. **(E)** DNA-dependent activator of interferon (DAI) can sense HSV likely through the recognition of HSV dsDNA and inhibit the activity of ICP0, leading to a decrease in viral genome replication. However, after DAI recognition downstream signaling events from STING, through NF-κB are blocked by the viral protein UL24. **(F)** The inflammasome is a multiprotein complex that assembles upon host sensor (e.g., AIM2, IFI16, NLRP3) encounter with viral determinants. The HSV protein VP22 has been reported to block AIM2 sensing of HSV and hence, block pro-caspase-1 activation by adaptor protein apoptosis-associated speck-like protein containing CARD (ASC). By blocking pro-caspase-1 activation, HSV inhibits the production of the pro-inflammatory cytokine IL-1β. Although the host sensor IFI16 has been reported to signal mainly through STING, it can also participate in inflammasome activation. **(G)** The HSV-2 protein ICP0 can direct IFI16 to degradation compartments, thus blocking downstream signaling events by this sensor. Black lines show cellular processes. Red lines show processes modulated by HSVs.

Besides TLRs, other host receptors also sense nucleic acids expressed during HSV infection, such as cytosolic retinoic-acid-inducible gene-1 (RIG-1)-like receptors and a broad class of putative DNA sensors (Mogensen, 2009). Importantly, viral nucleic acids can act as strong activators of host signaling pathways that lead to antiviral cellular responses (Iwasaki, 2012). Furthermore, the detection of viral nucleic acids frequently leads to the secretion of pro-inflammatory cytokines, as well as the production of IFNs that hamper viral replication and infection (Diner et al., 2015). Interferon-γ inducible protein 16 (IFI16) is a host sensor of nucleic acids that has been reported to be able to recognize episomal dsDNA, particularly DNA replicating in the nucleus of cells, which results in IFI16 acetylation (Ansari et al., 2015). This process is followed by the translocation of IFI16 to the cytoplasm, which leads to the promotion of IFN-β secretion by the cell and the activation of a host multiprotein complex called the inflammasome, able to initiate an inflammatory response (Unterholzner et al., 2010; Kerur et al., 2011). Importantly, HSV-1 and HSV-2 recognition by IFI16 induces the activation of the transcription factors interferon regulatory factor 3 (IRF3) and NF-κB, which once translocated to the nucleus induce IFN-α/β and IL-6 production in vaginal epithelial cells (Dawson and Trapani, 1995; Conrady et al., 2012; Triantafilou et al., 2014). IFI16 recognition of foreign DNA likely depends on the sensing of naked DNA. During HSV-1 infection, IFI16 may silence viral gene expression in human fibroblasts by adding nucleosomes and heterochromatin marks to the viral DNA, thereby restricting the host transcription machinery from accessing the viral genome (Orzalli et al., 2013). On the other hand, in epithelial cells the HSV-1 ICP0 protein has been reported to partially inhibit IFI16 activation by targeting it to the proteasome for degradation (Figure 3) (Johnson et al., 2013). A role for IFI16 in HSV infection has been assessed *in vivo*, with IFI16 knockdown mice unable to produce IFN-α and clear HSV-1 from the cornea after ocular infection (Conrady et al., 2012). Taken together, the studies described above indicate that HSVs readily modulate downstream pathways related to IFI16, as its activation seems to be detrimental to these viruses and their replication. Regretfully, to date only a few studies have assessed the roles of these sensors in immune cells in response to HSV infection.

cGMP-AMP synthase (cGAS) is a cytosolic DNA sensor that triggers cytosolic GMP-AMP (cGAMP) production upon binding to an activating DNA (Cai et al., 2014), cGAMP, in turn, acts as a messenger that signals through the transmembrane adaptor stimulator of interferon genes (STING) and leads to the recruitment and phosphorylation of TBK1, which ultimately activates IRF3-dependent production of IFN-α/β (Sun et al., 2013; Wu et al., 2013). Interestingly, HSV-1 recognition by cGAS leads to IFN-α and IFN-β secretion in fibroblasts, as well as immune cells (Orzalli et al., 2015). Furthermore, it has been shown that cGAS and IFI16 detect HSV cooperatively, with cGAS partially localizing in the nucleus and associating with IFI16 to promote the stabilization of the latter (Orzalli et al., 2015). Nevertheless, HSV-1 has been reported to be able to deregulate the function of these sensors. For example, the HSV-1 UL37 tegument protein has been shown to target cGAS and elicit its inactivation through the deamidation of an asparagine residue that is found both, in the human and mouse versions of this protein (Figure 3) (Zhang et al., 2018). In addition, apoptosis was observed following activation of cGAS after HSV-1 infection in human foreskin fibroblasts, which required cyclic dinucleotides and the activation of STING (Diner et al., 2016). On the other hand, protein kinase B (PKB, AKT) activation during HSV-1 infection has been observed to phosphorylate and suppress cGAS activity in epithelial cells, macrophages and fibrosarcoma cells *in vitro* (Figure 3) (Seo et al., 2015). The latter effect is likely due to HSV-1 US3 inhibiting Src family kinases and UL13-dependent VP11/12 tyrosine phosphorylation that leads to the modulation of the phosphatidylinositol-3 kinase (PI3K)/AKT signaling pathway (Eaton et al., 2014). Overall, PI3K/AKT modulation by HSVs would likely provide the virus the ability to interfere with cellular processes related to this pathway, such as cell metabolism, proliferation, gene expression, and cell survival (Liu and Cohen, 2015). Signaling through STING has been shown to be particularly important for conferring protection against ocular HSV-1 infection, as increased disease and virus replication were observed in the corneas and trigeminal ganglia of STING knockout mice, as compared to control animals (Parker et al., 2015). Consistently, treatment with 5,6-dimethylxanthenone-4-acetic acid (DMXAA), a STING agonist prior to infection protected mice from HSV neurological disease, which was associated with reduced viral replication thanks to increased type-I IFN production (Ceron et al., 2019).

Although cGAS is targeted early after infection by HSV, one wonders if it would be possible to detect this host sensor at later time points during cell infection. In this regard, the virion host shutoff protein (VHS, UL41) has been described to target cGAS for degradation even at 20 h post-infection, significantly reducing the chances that this receptor signal for IFN-β production upon HSV infection of epithelial cells and fibroblasts (Figure 3) (Su and Zheng, 2017).

Although some level of interference has been described by HSVs over the nucleic acid sensors described above, the DNA sensor termed DNA-dependent activator of interferon (DAI), which is expressed in primary vaginal tissue has been reported to readily detect HSV-2 and lead to IL-6 and IFN-β release upon infection (Triantafilou et al., 2014). This host sensor has been described to interact with the HSV-1 protein ICP0 to hamper viral genome replication, yet independent of the canonical DNA sensing function of this host factor (Figure 3) (Pham et al., 2013). Importantly, downstream events of the cGAS-STING signaling pathway, which are shared with those of DAI-STING, can be blocked by the HSV-1 serine protease UL24 protein that impairs NF-κB activation (Xu et al., 2017). Furthermore, VP24 can target TBK1 and hamper IRF3 phosphorylation, thus blocking alternative downstream signaling pathways associated with STING activation (Figure 3) (Zhang et al., 2016). Again, impairing IRF3 activation within infected cells will result in impaired IFN-I production and subsequent inhibition of interferon-stimulated genes (ISGs) in infected and neighbor cells.

RIG-1, as well as melanoma differentiation-associated protein 5 (MDA5), are two host sensors specialized in recognizing dsRNA (Weber et al., 2006). In the context of DNA viruses, such molecules are likely generated as byproducts during the transcription of viral genes and may derive from viral or host products, although this has not been reported for HSVs. Importantly, both receptors have been reported to have their signaling pathways modulated by the HSV protein VHS early after infection (Cotter et al., 2010; Yao and Rosenthal, 2011). This effect has been described to lead to impaired signaling events that otherwise should elicit IRF3 activation and an IFN-β-mediated antiviral response (Figure 3) (Yao and Rosenthal, 2011). RIG-1 has been reported to activate the STING pathway through an RNA-DNA sensor crosstalk mechanism aimed at restricting HSV-1 infection in epithelial cells and fibroblasts, as well as *in vivo* (Liu et al., 2016a). Additionally, the HSV-1 UL37 viral protein has been shown to directly block the function of RIG-1, through the deamidation of its helicase domain, which is needed for sensing dsRNA products (Figure 3) (Zhao et al., 2016).

In neuronal tissues DAI and RIG-1 work in tandem to detect HSV-1 in the CNS and elicit the production of the inflammatory cytokines TNF-α and IL-6 by murine glial cells, which altogether promote CNS inflammation and increased CNS permeability that allows immune cells to cross the blood-brain barrier, as well as IFN-I type-I to limit viral replication (Crill et al., 2015). Accordingly, RIG-1-mediated recognition of viral nucleic acids in this context depends on host DNA-dependent RNA polymerase III transcription of viral genes into mRNA harboring a 5′ triphosphate CAP structure, which is a substrate for RIG-1 and would allow an antiviral response either, directly or indirectly through DAI or RIG-1, respectively (Crill et al., 2015).

Another viral sensing pathway related to HSV and nucleic acids is the recognition of viral DNA and the activation of the inflammasome early after infection and then, its inhibition later during the virus replication cycle (Johnson et al., 2013). The inflammasome is a multiprotein complex composed by either one of the cytoplasmic sensors NLRP3 or AIM2, combined with IFI16 and has been described to sense HSV in keratinocytes (Chen and Ichinohe, 2015; Gimenez et al., 2016; Strittmatter et al., 2016). Consistent with this finding, a recent study found that IFI16 and NLRP3 are activated in human fibroblasts early after HSV infection (4 h) with consequent IL-1β release (Johnson et al., 2013). However, at later time points (8 h), IFI16 was found to be directed to the proteasome by the viral protein ICP0 and caspase-1, which is a pro-inflammatory effector induced by the inflammasome, and appeared to be trapped within actin clusters instead of being free in the cytosol to enact its catalytic activity (Figure 3) (Johnson et al., 2013). Additionally, HSV-1 has been reported to inhibit AIM2-dependent inflammasome signaling events by preventing its oligomerization through the viral protein VP22 (Maruzuru et al., 2018). Thus, HSVs also seem to have evolved molecular mechanisms to block the activation of the inflammasome within infected cells, as a mechanism to hamper the overall function of this sensor and therefore limit its effector capacity of alerting the cells of the presence of the virus.

Finally, virus-infected cells can also detect tertiary RNA structures derived from viral mRNAs thanks to protein kinase R (PKR), a host factor that once activated can help hamper the replication of viruses by inducing NF-κB activation and the expression of cytokines that control virus replication and infection (IFNs) (Kang and Tang, 2012). Furthermore, PKR can control protein synthesis by inducing its shutdown within the cell through the phosphorylation of the host translation initiation factor 2-alpha (eIF2α), which ultimately leads to cell apoptosis (Vattem et al., 2001). Because inhibition of translation within infected cells would be detrimental to the replication cycle of HSVs, these viruses override PKR function by inhibiting the phosphorylation of eIF2α thanks to the viral proteins γ34.5 and US11 (Figure 1) (He et al., 1997; Poppers et al., 2000; Carr et al., 2005). Thanks to these viral factors, HSVs can bypass cellular processes elicited after contact of host sensors with viral nucleic acids to enable productive viral infection and virus replication within infected cells.

Taken together, several nucleic acid receptors other than TLRs can sense activating nucleic acids generated during HSV infection. The recognition of such ligands likely helps counteract virus infection and dissemination to other cells and tissues within the host. Importantly, several of these receptors are known to recognize dsRNA structures, yet the origin of these nucleic acids in the context of HSV infection has not been established, and further studies are needed for their identification.

## HSVs Interfere With the Host Interferon Response

The activation of pathogen recognition receptors (PRR), can lead to the activation of immune and non-immune cells and trigger antiviral responses that restrict and interfere with virus replication. A significant antiviral response elicited by the sensing of viruses is the IFN response. IFNs are cytokines that once bound to their receptor can potentiate antiviral activities both, in the cell that secretes these molecules and neighbor cells (Schoggins, 2014). IFNs are classified as type-I, -II, or -III. Type-I IFNs are a broad family of molecules that can be secreted by numerous cell types early after infection in response to pathogens such as viruses, with some well-known members being IFN-α, IFN-β, and IFN-ε, and others more recently described IFN-υ, IFN-ω, and IFN-ζ (Hemmi et al., 2002; Al-Khatib et al., 2004; Diebold et al., 2004; Oritani and Tomiyama, 2004; Theofilopoulos et al., 2005; Ma et al., 2018). On the other hand, type-II IFNs have a sole family member, namely IFN-γ which is secreted by specialized subsets of immune cells usually late during infection (Boehm et al., 1997; Bigley, 2014). Finally, type-III IFNs such as IFN-λ1, IFN-λ2, and IFN-λ3 are usually secreted early during infection and have somewhat similar effects than type-I IFNs, although their secretion is limited to epithelial cells (Donnelly and Kotenko, 2010). While type-I and type-III IFNs are related to the induction of multiple antiviral effects in several cell types, type-II IFNs are more related to regulatory roles among immune cells and are accordingly mainly expressed by such types of cells, such as T helper cells (Tau and Rothman, 1999).

**Figure 4:**
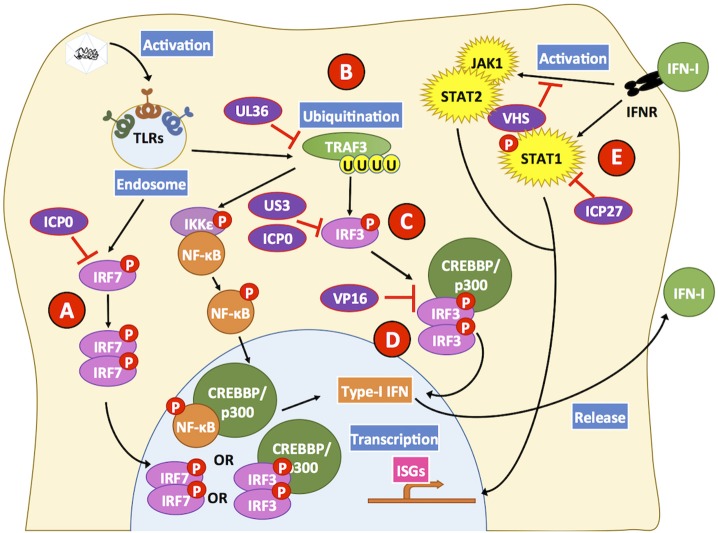
HSV proteins modulate key steps in interferon-related pathways. HSV proteins inhibit interferon-related pathways. Engagement of Toll-like receptors (TLRs) by viral determinants leads to the activation of transcription factors that induce the expression of type-I IFNs. **(A)** The HSV ICP0 protein can block IRF7 activation by hampering its phosphorylation and consequently inhibit its translocation to the nucleus. **(B)** Additionally, UL36 inhibits the ubiquitination of TRAF3 which is required for positive downstream signaling and activation of the transcription factors NF-κB and IRF3. **(C)** HSV proteins US3 and ICP0 can interfere with IRF3 activation at this stage, thus blocking this signaling pathway that otherwise would lead to type-I IFN expression. **(D)** Furthermore, VP16 inhibits the formation of the IRF3-CREBBP/p300 complex hampering signaling events that would lead to IFN-I expression. **(E)** Upon IFN-I engagement, IFNR on the cell surface elicits intracellular signaling cascades mediated by STAT1, STAT2, and JAK1. However, the viral protein ICP27 interferes with STAT1 activation and the viral protein VHS hampers STAT2- and JAK1-related signaling pathways that otherwise would induce the expression of ISGs, which elicit antiviral effects. Black lines show cellular processes. Red lines show processes modulated by HSVs.

Because IFNs have detrimental effects on viruses, HSVs encode an array of molecular factors that negatively modulate the induction of IFN, their production, secretion, and their associated effects by interfering, among others with their intracellular signaling pathways (Peng et al., 2009). For example, the ICP0 proteins of both, HSV-1 and HSV-2 have been described to directly bind and interfere with the activation of IRF3 and IRF7, two transcription factors related to the expression of type-I IFNs (Figure 4) (Eidson et al., 2002; Lin et al., 2004; Zhang et al., 2015). Importantly, mice that lack both IRF3 and IRF7 (IRF3/7 double knockout mice) have been described to suffer increased HSV-1 replication and display enhanced dissemination of this virus to several organs after corneal infection (Murphy et al., 2013). Additionally, the HSV-1 US3 protein has been reported to hyperphosphorylate IRF3, which impairs its dimerization and nuclear translocation, thus hampering the transcription of *IFNB* mediated by this transcription factor (Wang et al., 2013b). Furthermore, the HSV-1 tegument protein VP16 has been shown to block IFN-β expression through the inhibition of IRF3 and NF-kB, by impairing the recruitment of the shared coactivator CREB binding protein (CBP) to IFN-I promoters, which is required by these transcription factors to induce IFN-I expression (Figure 4) (Xing et al., 2013). Accordingly, the HSV-1 protein UL36, an ubiquitin-specific protease has been shown to de-ubiquitinate TRAF3 (TNF receptor-associated factor-3), thereby hampering stimuli-induced IRF3 dimerization, which is required for IRF3 translocation to the nucleus thus, inhibiting IFN-β transcription (Figure 4) (Wang et al., 2013a). Moreover, the advantage for HSVs in interfering with the signaling events associated to type-I IFN secretion has been evidenced *in vivo*, as low IFN-α and IFN-β production is observed in the genital tract of mice after infection (Milligan and Bernstein, 1997; Peng et al., 2009).

When IFNs are released from infected cells, they can act either as paracrine or autocrine ligands by binding to IFN receptors on the cell surface and induce ISG within cells. Antiviral effects of IFNs include restricting the replication of the viral genome, inhibition of protein translation, and impaired virus egress (Schoggins and Rice, 2011). In order to counteract these outcomes, HSVs interfere with signaling events that occur downstream of the IFN receptors. For instance, the HSV-1 ICP27 protein affects STAT-1 activation, which is a signal transducer for ISG transcription. ICP27 has been reported to interfere with the phosphorylation and nuclear accumulation of STAT-1 in order to impair its activity as a transcription factor (Figure 4) (Johnson et al., 2008). Additionally, there is indirect evidence, through an HSV-1 mutant, that suggests that the viral protein VHS could partially be responsible for reducing the activity of signal transducers such as JAK1 and transcription factors like STAT-2, as observed in HSV-infected HeLa cells (Figure 4) (Chee and Roizman, 2004). Additionally, the HSV-1 ICP27 protein has been reported to be involved in the secretion of an uncharacterized soluble factor that has antagonizing activity over IFN-I signaling pathways in neighboring uninfected cells (Johnson and Knipe, 2010). *In vivo* studies have assessed the relevance of IFN-I in HSV infection in mice that lack the receptors for type-I IFNs, namely IFNAR1 and IFNAR2c, and shown that inoculation of HSV-1 in the footpads of such animals results in a reduced capacity of the host to control HSV replication, leading to systemic infection, although non-lethal (Luker et al., 2003).

On the other hand, IFN-α*βγ*R^−/−^ mice have been found to be highly susceptible to acute liver failure after HSV-1 corneal infection, with IFN-α*βγ*R expression in both, immune and non-immune cells playing relevant roles in the control of systemic HSV infection (Pasieka et al., 2011; Parker et al., 2016). Moreover, a key role for type-I IFN signaling has been identified in neurons, since immune cell and non-neuronal cell IFN responses do not protect from lethal corneal HSV infection when the these pathways are abrogated in neurons (Rosato and Leib, 2015). In a later study, it was found that IFN-I signaling in neurons was dispensable for the establishment of latency and that cells deficient in IFN-I signaling supported reduced reactivation yet, displayed higher levels of LAT indicating that IFN-I likely regulates LAT expression in neurons (Rosato et al., 2016). Consistent with the relevance of type-I IFNs in HSV infection, topical application of IFN-α was reported to significantly reduce the frequency of recurrences and viral shedding in patients suffering from genital HSV reactivations (Shupack et al., 1992). Although HSVs have mechanisms to impair type-I IFN secretion and their effects, such molecules may eventually reach adjacent cells that are non-infected and elicit signaling events in these cells (Gill et al., 2011; Lee et al., 2017).

IFN-γ induction is associated with positive outcomes during HSV-1 and HSV-2 infections, with reduced viral replication. Furthermore, IFN-γ may be considered a marker related to the potential efficacy of prophylactic formulations (Svensson et al., 2005; Bird et al., 2007; Sato et al., 2014; Khan et al., 2015). Without IFN-γ, T cells are incapable of conferring protection against HSV genital infection (Johnson et al., 2010). However, the relationship between IFN-γ and HSV control is intricate, as the antiviral effects of this cytokine are tissue-dependent and vary depending on whether the virus remains latent in infected cells or is productive in the lytic cycle (Bigley, 2014). Among numerous effects, IFN-γ causes microtubule remodeling in infected cells, which is mediated through the activity of the molecules suppressors of cytokine signaling 1 and 3 (SOCS1 and SOCS3). However, elevated SOCS expression elicits microtubule stabilization and an inhibition feedback on IFN-γ effects, which has been exploited by the HSV-1 ICP0 protein, capable of upregulating SOCS during lytic infection in keratinocytes (Frey et al., 2009). Although IFN-γ acts over promoters of IFN-γ-stimulated genes (ISGs) that have antiviral functions, ISG expression is restricted by epigenetic regulations of histone 3 (H3) in the trigeminal ganglia and is dependent on histone deacetylases (HDACs) to maintain chromatin in a transcriptionally inactive state (Gao et al., 2013). During HSV-1 infection of the trigeminal ganglia, neurons may respond to stress stimuli (e.g., UV light) and inhibit HDACs, which results in SOCS1 and SOCS3 acetylation and the loss of IFN-γ effects. Additionally, chromatin may suffer relaxation processes allowing the viruses to exit latent infection of neurons (Guise et al., 2013). Although a relevant role for IFN-γ has emerged from some studies, paradoxically mice lacking IFN-II receptors IFNGR1 and IFNGR2 showed comparable levels of viral loads as controls when challenged with HSV-1, suggesting that the effects of IFN-γ are somewhat complex in the context of HSV infection (Luker et al., 2003).

Regarding type-III IFNs, relatively few studies have assessed their role during HSV infection. However, one study has reported that the administration of IFN-λ1 (IL-29) prior to HSV-1 infection promoted the expression of numerous antiviral proteins in primary human keratinocyte cultures. One of them, IFN-β helped prevent their infection. This effect was dependent on TLR3 engagement and JAK-STAT signaling events (Zhang et al., 2011). Furthermore, in human neurons HSV-1 infection was shown to be suppressed by IFN-λ1 and IFN-λ2 (IL-28A), particularly through the upregulation of TLR3 and TLR9 expression and subsequent TLR3/9-mediated antiviral responses involving the transcription factor IRF7 (Zhou et al., 2011). Interestingly, type-III IFNs have been reported to be secreted in the vaginal mucosa mainly by DCs, yet if this is the case during HSV infection remains to be determined (Iversen et al., 2010).

Taken together, HSVs have evolved several mechanisms to interfere with the host IFN response at multiple levels. Indeed, HSVs can impair IFN secretion and their related signaling events in infected cells. Collectively, the capacity of HSVs to interfere with IFN responses at various steps highlights the importance of these molecules and pathways in HSV control. Unsurprisingly, potential therapeutic approaches, such Imiquimod induce type-I IFN secretion (Sainathan et al., 2012).

## HSVs Down-modulate the Antiviral Activities of the Complement and Innate Immune Cells

If HSV-infected cells are unable to restrict the replication of these viruses or their dissemination, an innate immune response consisting on both acellular and different cell types, will likely interact with the viruses or virus-infected cells in an attempt to impede further infection of nearby cells or other tissues (Halford et al., 2005; Nandakumar et al., 2008; Tegla et al., 2011).

However, HSVs are able to inhibit the chain reactions carried out by the host complement which is intended to hamper pathogens by initiating a cascade of protein activations that lead to a membrane attack complex (MAC) (Serna et al., 2016). Indeed, the gC glycoprotein of HSVs can bind to the complement component C3b and block alternative pathways that otherwise lead to the formation of a MAC on the pathogen surface, or the surface of virus-infected cells (Friedman et al., 1984; Mcnearney et al., 1987. Additionally, gC also binds to the complement components C3 and C5, further inhibiting pathways related to the activation of this antiviral mechanism (Lubinski et al., 2002; Hook et al., 2006b).

**Figure 5:**
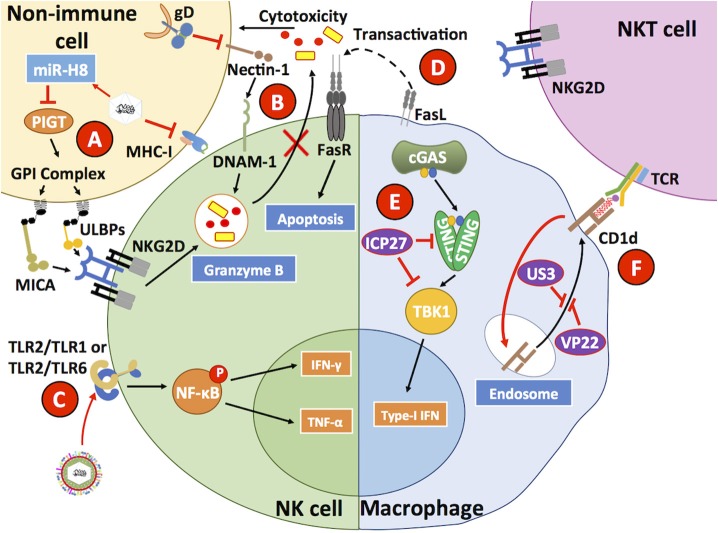
HSVs interfere with antiviral processes in innate immune cells. **(A)** HSV has been described to reduce MHC-I expression on the surface of infected cells. In addition, HSV also reduces MICA and ULBP1-3 expression through the inhibition of PIGT, a member of the GPI anchoring complex by the HSV-1-encoded microRNA H8 (miR-H8). **(B)** The HSV glycoprotein gD reduces nectin-1 expression on the surface of infected cells, hampering DNAM-1 binding to this host factor and diminishing the capacity of NK cells to mediate the lysis of HSV-infected cells, which is normally mediated by granzymes. **(C)** HSV has been reported to directly engage TLR2 on the surface of NK cells, which leads to IFN-γ and TNF-α secretion. **(D)** FasL expressed on the surface of HSV-infected macrophages has been reported to induce Fas-mediated apoptosis in NK cells. **(E)** Within HSV-infected macrophages, the HSV protein ICP27 has been reported to inhibit STING and TBK1 activation, thus interfering with this signaling pathway that generally leads to IRF3-dependent type-I IFN secretion by virus-infected cells. **(F)** HSV infection of macrophages reduces the surface expression of CD1d, which in combination with a glycolipid acts as a receptor for NKT cell TCRs. CD1d is sequestered by the HSV proteins US3 and VP22. Thus, HSV reduces NKT expansion and function by hiding its activating ligand. Black lines show cellular processes. Red lines show processes modulated by HSVs.

On the other hand, natural killer (NK) cells are innate immune cells capable of sensing and destroying virus-infected cells that either lack the expression of major histocompatibility complex I (MHC-I) molecules, or express NK-activating molecules on the surface because of abnormal cellular processes betray infection (Mandal and Viswanathan, 2015). HSVs hampers MHC-I expression on the surface of infected cells, which under normal conditions should elicit the activation of NK cells (Orr et al., 2005). However, HSV-1 infection has been shown to reduce the expression of MHC class I polypeptide-related sequence A (MICA) and UL16 binding proteins 1–3 (ULBP1, ULBP2, ULBP3) on the surface of infected cells, which are activators of NK cells that mediate signaling events through the engagement of NKG2D in these cells (Figure 5) (Schepis et al., 2009). This inhibition has been reported to be mediated by HSV-1-encoded mir-H8, which downregulates PIGT expression, a member of the GPI anchoring complex that anchors MICA and ULBP1-3 and results in the surface downregulation of these NK ligands (Enk et al., 2016). Therefore, NK cells do not release cytotoxic molecules, such as granzymes onto HSV-1-infected cells, protecting these cells from NK-mediated apoptosis. Nevertheless, NK cells pulsed with HSV-1 and HSV-2 glycoprotein gD antigens and inoculated with TLR2 agonists produced IFN-γ that activated antiviral CD4^+^ T cells (Kim et al., 2012). Consistent with immune evasion properties by HSVs, the HSV-1 gD glycoprotein has been reported to sequester nectin-1 from the cell surface of infected cells and induce decreased DNAX accessory molecule-1 (DNAM-1) receptor engagement on the surface of NKs by this ligand, thus preventing NK cell-mediated lysis of infected cells (Figure 5) (Grauwet et al., 2014). Finally, macrophages infected with HSV-1 have been described to express FasL and induce apoptosis in NK cells that express Fas receptors (Iannello et al., 2011).

Another innate immune cell type known to participate in antiviral responses is Natural Killer T cells (NKT cells). NKTs recognize antigens in the form of glycolipids presented on CD1d molecules that share structural similarities with MHC-I (Godfrey et al., 2010). Importantly, HSV-1 has been described to negatively affect NKT activation by downregulating CD1d expression on the surface of infected cells (Figure 5) (Yuan et al., 2006; Rao et al., 2011). More specifically, HSV-1 was shown to redirect CD1d from the cell surface to intracellular compartments through the phosphorylation of the host factor KIF3A by the viral kinase US3 (Xiong et al., 2015). Furthermore, cellular recycling of CD1d was also inhibited by the viral protein VP22 working along US3 (Liu J. et al., 2016). Importantly, the administration of α-galactosylceramide, an NKT ligand that elicits the recruitment of these cells to the vaginal tissue was reported to decrease the susceptibility of mice upon HSV-2 intravaginal infection (Iversen et al., 2015).

Macrophages are also targets of HSVs. In these cells, HSV-1 has been reported to inhibit downstream events related to the cGAS-STING-TBK1 axis, particularly through the direct interaction of ICP27 with STING and TBK1, which produced a reduction in IFN-I secretion by these cells (Figure 5) (Christensen et al., 2016). Interestingly, STAT-1-knockout mice, which are unresponsive to IFN-α and IFN-γ, have been found to be more susceptible to HSV-1 in terms of macrophage infection, as compared to wild-type mice suggesting that these cells utilize a JAK-STAT-1 signaling pathway to restrict HSV replication (Mott et al., 2009). Additionally, HSV-1 has been shown to produce higher levels of pro-inflammatory cytokines in M1 macrophages as compared to M2 macrophages, with M1 characterized as “classically polarized” macrophages vs. M2 macrophages that are “alternatively polarized” (Martinez and Gordon, 2014). The latter observation suggests that pro-inflammatory M1 macrophages infected by HSV-1 promote increased eye inflammation (Lee and Ghiasi, 2017). On the other hand, in the same study when macrophages were stimulated to induce their differentiation toward an M2 phenotype, these cells produced anti-inflammatory cytokines (e.g., IL-10), which was associated with less eye pathology.

Regarding other innate immune cell types, such as neutrophils or mast cells, these cells have been described to participate at the onset of immune cell infiltration into skin and corneas during HSV infection (Royer et al., 2015; Hor et al., 2017). However aside from contributing to exacerbated inflammation, a protective role has not been attributed to neutrophil activity in these tissues in mice models, yet mast cells seem to be necessary for assisting innate immunity in the cornea of mice (Royer et al., 2015; He et al., 2017).

Altogether these results suggest that HSVs target NK and NKT cells, as well as macrophages because these cells likely play a crucial role in controlling HSV infection. Thus, potentiating the activation and functions of these cells during HSV exposure and infection could elicit improved responses against these viruses.

## HSV Infection Modulates Dendritic Cell Maturation, Antiviral Activity and Migration

Dendritic cells (DCs) are key immune cells that promote and regulate immune responses by modulating the activity of innate and adaptive immune cells (Gonzalez et al., 2008; Cespedes et al., 2013). DCs are strategically located throughout the body acting as sentinels that probe the environment surrounding mucosae, skin, as well as internal organs. Ultimately, DCs sense and capture foreign and self-antigens for their processing (Soloff and Barratt-Boyes, 2010). DCs degrade protein-derived antigens and present them to T cells as small peptides loaded on MHC-I and -II molecules (pMHC) that can be recognized by T cell receptors (TCR) on the surface of CD8^+^ and CD4^+^ T cells, respectively (Galvez et al., 2016). DC antigen presentation to T cells can lead to a process termed the immunological synapse, which involves close DC-T cell interactions that can result either in T cell activation or its inactivation (Gonzalez et al., 2007; Murphy et al., 2012; Retamal-Diaz et al., 2015; Retamal-Diaz A. et al., 2017). Importantly, the interaction between DCs and antigen-specific T cells will determine the phenotype of T cells which will depend on the expression of membrane-bound and soluble molecules presented at the cell-cell interphase (Zheng et al., 2004). As a result of DC-T cell activation, T cells can become among other cell types, cytotoxic or regulatory by secreting soluble factors that kill infected cells, modulate immune, and non-immune cells, or promote tolerance to antigens, eventually ignoring cognate antigens (Gonzalez et al., 2007).

**Figure 6:**
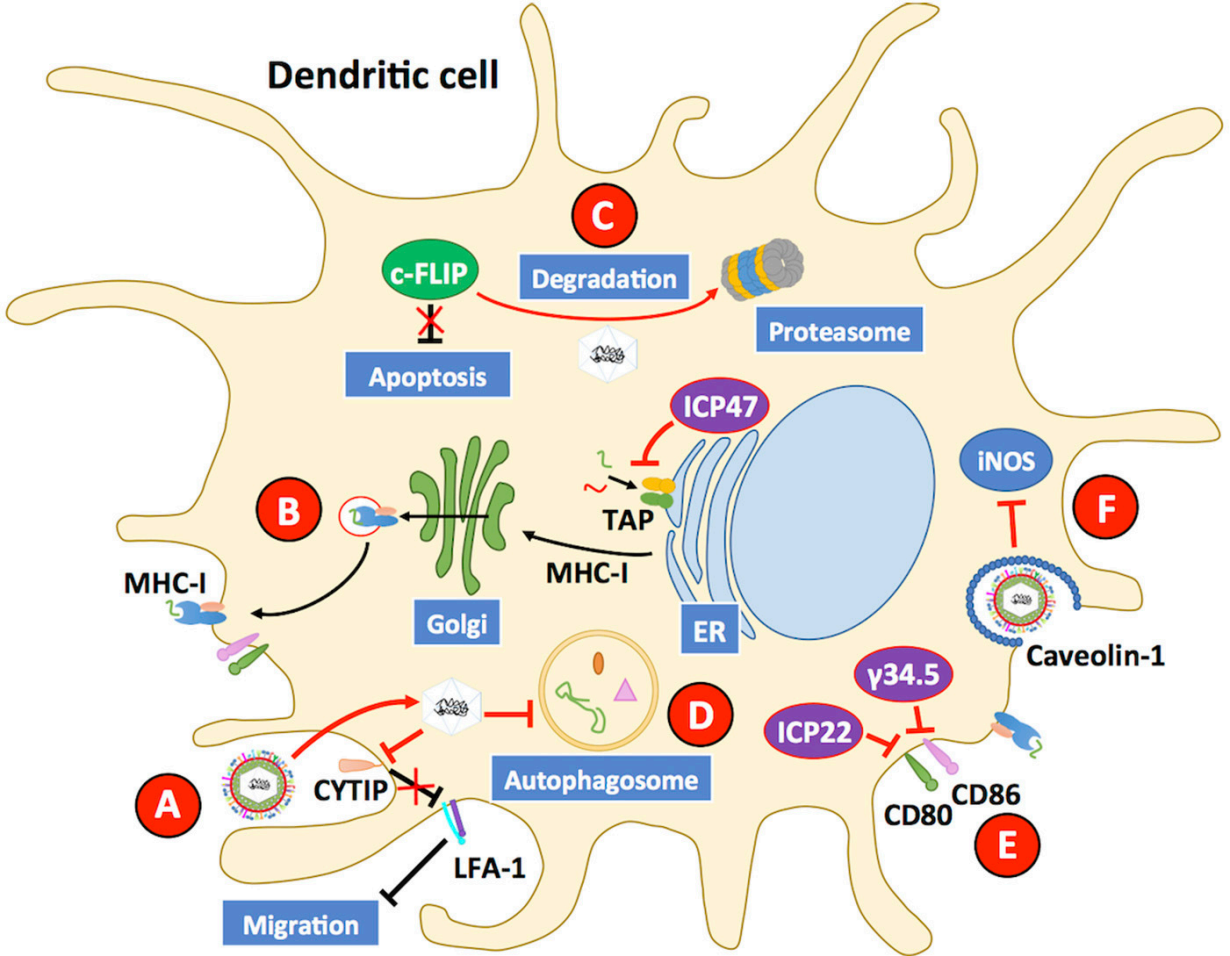
HSVs interfere with dendritic cell function. Dendritic cells (DCs) are susceptible to HSV-1 and HSV-2 infection. **(A)** Upon infection with HSV, the host protein CYTIP is degraded, which causes the upregulation of LFA-1 and reduces the capacity of DCs to migrate to draining lymph nodes and activate T cells. **(B)** HSV infection hampers the capacity of DCs to present virus-derived antigens to T cells on MHC-I molecules by interfering with the activity of transporters associated with antigen presentation (TAP proteins). Inhibition of TAPs is mediated by the viral protein ICP47. **(C)** HSVs elicit apoptosis in DCs through the downregulation of c-FLIP, a potent anti-apoptotic protein, which is directed to the proteasome during infection of these cells. **(D)** HSV infection hampers the activity of the autophagosome, which has been reported to reduce antigen presentation to CD8^+^ T cells. **(E)** CD80 and CD86 are co-stimulatory molecules that are commonly upregulated during infection, and along with MHC-peptide complexes enable DCs to activate T cells. HSV inhibits the expression of CD80 and CD86 on the DC surface thanks to the viral proteins γ34.5. The viral protein ICP22 also inhibits the expression of CD80 on the cell surface. **(F)** HSV infection inhibits inducible nitric oxide synthase (iNOS) in DCs through the downregulation of caveolin-1, which will reduce the antiviral capacity of these cells. Black lines show cellular processes. Red lines show processes modulated by HSVs.

Because of the role of DCs in defining the phenotype of T cells, which in turn can affect the overall immune response against a viral pathogen such as HSV, the interaction between DCs and these viruses has gained increasing attention in the last decade. Importantly, DCs are permissive to HSV infection, although virus yields are somewhat limited as compared to other cellular substrates, such as epithelial cells (Pollara et al., 2003; De Jong et al., 2008; Grosche et al., 2017; Retamal-Diaz A. et al., 2017). Once infected with HSVs, DCs display reduced antigen presentation on MHC-I molecules, which is mediated by the viral protein ICP47 that acts over transporters associated with antigen processing (TAP) at the endoplasmic reticulum and impedes antigen translocation to this organelle for the loading of viral antigenic peptides onto MHC-I molecules (Figure 6); yet, this phenomenon has been reported to occur at a lower extent in murine cells, as compared to human cells (Hill et al., 1995; Tomazin et al., 1998; Elboim et al., 2013; Oldham et al., 2016a). Interestingly, ICP47 has been reported to adopt a helical hairpin structure that blocks TAP function and peptide translocation, as it precludes substrates from binding to the transporter and prevents the two cytoplasmic nucleotide-binding domains (NBD) of TAP from hydrolyzing ATP, which is required for their activity (Oldham et al., 2016b). Despite the existence of several variants of TAP-1 and TAP-2 in humans, ICP47 does not seem to have a particular preference over one or other polymorphism (Praest et al., 2018).

HSV-1 and HSV-2 can also reduce the capacity of DCs to activate T cells by decreasing the expression of the co-stimulatory molecules CD80 and CD86 on the cell surface, which has been suggested to occur through the downregulation of IFNα/β levels by the viral protein γ34.5 (Figure 6) (Jin et al., 2009; Suazo et al., 2015a). Consistently, an HSV-1 with a mutation in γ34.5 is capable of inducing the maturation of DCs through TBK-1-dependent phosphorylation of IRF3 (Ma et al., 2017). However, a later study suggests that inhibition on IRF3 activation by γ34.5 is also mediated by mechanisms other than TBK-1, as the deletion of the TBK-1 binding domain (TBD) of γ34.5 did not restore IRF3 activation, although this finding remains to be confirmed in DCs as the study was performed in human foreskin fibroblasts cells (Manivanh et al., 2017). On the other hand, the HSV-1 protein ICP22 has been reported to be capable of binding to the CD80 promoter in DCs circulating through HSV-infected cornea, inhibiting the expression of this important co-stimulatory molecule for T cells (Matundan and Ghiasi, 2018).

Moreover, both HSV-1 and HSV-2 have been reported to inhibit autophagosome formation in DCs, by interfering with cellular degradation processes and affecting antigen presentation to CD8^+^ T cells (Suazo et al., 2015a; Budida et al., 2017). Because DCs utilize autophagy as a means to limit viral replication within these cells, inhibition of this process likely contributes to HSV subversion of DCs (Figure 6) (Rasmussen et al., 2011). HSV-1 has been described to interfere with nitric oxide synthase within lung DCs via downregulation of caveolin-1 (Cav-1), further hampering the antiviral capacities of HSV-infected DCs (Figure 6) (Wu et al., 2015).

Additionally, HSV-1 and HSV-2 have been reported to hamper DC migration from the infected tissue to the corresponding lymph nodes (LNs), thereby likely reducing the efficacy of DCs at activating CD4^+^ and CD8^+^ T cells at this site (Prechtel et al., 2005; Bedoui and Greyer, 2014; Retamal-Diaz A. et al., 2017). Indeed, HSV-1 has been shown to promote the degradation of cytohesin-interacting protein (CYTIP) in mature DCs, which regulates DC motility by downregulating integrin expression and causes the upregulation of lymphocyte function-associated antigen-1 (LFA-1), a β2-integrin protein; therefore enhanced adhesion of DCs occurs in the infected tissue reducing their migration to the LNs (Figure 6) (Theodoridis et al., 2011). Additionally, HSV-1- and HSV-2-infected Langerhans cells (LCs) have been reported to undergo apoptosis after infection with HSVs and to be unable to downregulate E-cadherin, which needs to be reduced at the cell surface to promote the migration of these cells to the LNs (Puttur et al., 2010). In this context, HSV-infected LCs have been described to act as a source of HSV antigen for dermal DCs (dDCs) within the infected skin, which would result in the phagocytosis of apoptotic HSV-infected LCs by dDCs (Kim et al., 2015). The interaction between LCs and HSV, and then by HSV-infected and apoptotic LCs with dDCs would likely result in the priming of HSV-specific T cells *in vivo*, which would be difficult to assess *in vitro* with HSV-infected bone marrow-derived DCs (BMDCs) and monocyte-derived DCs. Importantly, the effects of HSV infection over the capacity of DCs to activate T cells seems to more pronounced *in vitro* than *in vivo* (Bedoui et al., 2009; Kim et al., 2015; Whitney et al., 2018).

However, contrarily to the negative effects described above for HSV over DCs, another study found that upon exposure to HSV-1, a human CD8α^+^ plasmacytoid DC subset increased the expression of markers associated with the migration of these cells to lymph nodes, and that these DCs were able to promote the activity and functions of T cells, B cells and NK cells, which were recruited to the infection site (Schuster et al., 2015).

Overall, most of the findings described above support the notion that HSVs have evolved different mechanisms and strategies to hamper DC function impacting virus control by these cells and likely negatively affecting adaptive immune responses in the host.

Despite numerous studies describing approaches that elicit protective immunity against HSVs, identifying a correlate of protection for HSV infection has remained elusive. Interestingly, recent studies suggest that the outcome of the DC-HSV interaction may relate to the establishment of protective immunity, as specific HSV mutants that are attenuated in DCs confer particularly protective and robust immunity against HSV infection *in vivo* (Retamal-Diaz A. et al., 2017; Retamal-Diaz A. R. et al., 2017). One of these studies reported that anti-HSV antibodies mediated the protection conferred by the HSV-inoculated DCs, which likely results from the help of B cell-supportive anti-HSV helper T cells (Long et al., 2014). On the other hand, vaginal DCs primed with estradiol have been described to promote CD4^+^ T cells with a Th17 profile that enabled these cells to efficiently respond against an HSV-2 challenge (Anipindi et al., 2016). An IL-1β-related signaling pathway mediated this favorable response. The relevance for DCs in eliciting protective anti-HSV responses has been further emphasized by studies that assess their contribution at re-stimulating tissue-resident memory T cells (T_RM_) (Iijima et al., 2008). After HSV-2 infection, T_RM_ CD8^+^ are recruited to the genital tissue by chemokines such as CXCL-9 and CXCL-10, which are expressed by the infected epithelium (Nakanishi et al., 2009; Iijima and Iwasaki, 2014). Importantly, this recruitment was found to be mediated, at least partially by IFN-γ produced by DCs which came into contact with HSV antigen-specific Th1 helper CD4^+^ cells and stimulated them to establish T_RM_ CD8^+^ cells (Smith et al., 2004; Nakanishi et al., 2009). In line with this notion, a “prime and pull” immunization approach was recently described with which protective immunity was achieved against HSV-2 genital infection upon inoculation of an attenuated HSV virus which induced vaginal tissue memory T cells that could be recalled to this tissue in a CXCL10-dependent manner (Shin and Iwasaki, 2012). dDC populations present in the skin within the CD301b^+^ subset were found to be present at the site of infection after applying “prime and pull” strategy mentioned above and were held responsible for re-stimulating HSV antigen-specific memory CD8^+^ T cells (Shin et al., 2016).

HSVs have also been reported to induce the synthesis and release of pro-inflammatory cytokines by DCs that promote their infection with HIV and the replication of this virus from previously-infected cells, likely increasing the dissemination of the latter virus during co-infections (Stefanidou et al., 2013a). HSV-2-infected DCs secrete TNF-α, which through signaling processes mediated by TNFR1 and TNFR2 has been reported to induce increased expression of CCR5 in DCs, enabling subsequent infection of these cells with HIV-1 (Marsden et al., 2015; Herbring et al., 2016).

Recent studies support the notion that DCs may promote neuron infection with HSV, thus contributing to virus latency within the host. In addition, it has been observed that animals depleted of DCs display up to fivefold less latently infected neurons in the trigeminal ganglia, as compared to wild-type mice suggesting that DCs participate in processes related to neuron infection (Mott and Ghiasi, 2008). Accordingly, the depletion of the CD11c^+^CD8α^+^ DC subset reduced the amounts of latent HSV-1 in neurons after ocular infection (Mott et al., 2009). Furthermore, Flt3L treatment, which increases the numbers of DCs in tissues, produced increased neuronal infection upon a similar infection (Mott et al., 2008). Taken together these studies suggest HSV may use DCs as Trojan horses to reach neurons, which may occur by virus attachment to the cell surface or virus replication within these cells. Despite these findings, another study found that depleting DCs with diphtheria toxin targeting CD11c-expressing cells was associated with increased viral loads in neurons after HSV infection in the footpads (Kassim et al., 2006). Another study found that mice lacking CD8α^+^ DCs had increased amounts of latent HSV-1 and more recurrences (Mott et al., 2014).

Overall, HSVs have evolved multiple mechanisms to negatively modulate the function of DCs, which likely results in a reduced capacity of the host to control HSV infection and mount and effective antiviral response. Identifying strategies that improve the interaction between HSVs and DCs should likely ameliorate the overall host response to these viruses either, immediately upon infection or during the establishment of long-term protection.

## Concluding Remarks

Herpes simplex viruses elicit a diverse array of diseases in humans, both in individuals that have immune-related complications, as well as otherwise healthy persons. The capacity of HSVs to elicit disease during primary infections, as well as recurrences after establishing lifelong infection relates, among others to their ability to evade and neutralize host antiviral mechanisms that act in immune and non-immune cells. Importantly, HSVs interfere with early antiviral steps, such as the capacity of the host to sense viral determinants, signaling pathways that lead to cellular antiviral effects and the function of innate immune cells that act early after infection against these viruses. Evasion of these processes gives HSVs the chance to infect host cells and reach neurons favoring viral latency and lifelong infections, altogether dampening antiviral activities that could help immune cells establish effective and protective immunity against these viruses. The fact that the host somewhat fails at initiating an effective early antiviral response may provide grounds for the establishment of ineffective adaptive immunity, mainly through the interference of DC function, which is crucial for linking innate and adaptive immunity. Thus, improving the outcome of the early host antiviral responses against HSVs could help both, the generation of better anti-HSV therapies, as well as the design of prophylactic strategies intended at preventing infection with these viruses.

## Author Contributions

All authors listed have made substantial, direct and intellectual contributions to the work, and approved it for publication.

### Conflict of Interest Statement

The authors declare that the research was conducted in the absence of any commercial or financial relationships that could be construed as a potential conflict of interest.
